# Generations in the grove: the Dongria Kondh's ecological kinship and intergenerational resilience

**DOI:** 10.3389/fsoc.2026.1741022

**Published:** 2026-05-21

**Authors:** Anjali Yadav

**Affiliations:** Faculty of Law, University of Delhi, New Delhi, India

**Keywords:** Dongria Kondh, indigeneous knowledge, intergenerational solidarity, sustainability, traditional ecological knowledge

## Abstract

This article repositions the family as a central yet overlooked institution in environmental governance by examining the Dongria Kondh of the Niyamgiri hills in India. Moving beyond dominant policy narratives that treat families as passive beneficiaries of sustainability frameworks, the paper argues that indigenous households function as sites where ecological knowledge, legal agency, and intergenerational solidarity converge to produce enduring forms of environmental stewardship. Drawing on a critical interpretive review of statutory law, constitutional jurisprudence, ethnographic accounts, and policy documents, the study develops the concept of familial ecological sovereignty to capture how kin-based institutions sustain ecological continuity through everyday practices of care, ritual, and decision-making. The analysis situates Dongria Kondh familial practices within India's plural legal landscape, particularly the Forest Rights Act, 2006, and relevant Supreme Court jurisprudence, including the landmark Niyamgiri case. It demonstrates that legal consciousness is not solely generated in courts or formal institutions but is cultivated within domestic spaces through intergenerational transmission of norms, values, and ecological understanding. At the same time, the paper critically engages with internal tensions within families, including gendered authority, migration, and intra-community inequalities, cautioning against idealized portrayals of indigenous governance. By integrating insights from legal pluralism, relational sociology, and traditional ecological knowledge, the study advances a conceptual triad linking legal agency, ecological knowledge, and intergenerational solidarity as mutually reinforcing processes. It argues that sustainability in indigenous contexts is not merely a matter of resource management but a relational practice rooted in kinship and moral obligation across generations. The paper concludes by proposing a shift in environmental governance from technocratic and centralized models toward relational frameworks that recognize families as co-governors of ecological systems. It outlines modest but actionable legal and policy reforms within existing statutory structures to better accommodate familial forms of knowledge and authority. In doing so, the study contributes to broader debates on decolonizing sustainability and foregrounds the role of intergenerational solidarity in shaping resilient ecological futures.

## 1 Introduction

If we look across the world, indigenous families have long served as custodians of ecological knowledge and moral responsibility for the land they inhabit. Even in the Indian context, the continuity of numerous forest-dwelling communities hinges on the robustness of kinship systems that perpetuate environmental values, ceremonial duties, and essential skills through successive generations.

The Dongria Kondh, an Adivasi group residing in the Niyamgiri hills of Odisha, exemplify this interweaving through practices such as communal forest management, traditional ecological knowledge (TEK) transmission, and rituals honoring nature. They regards the forest not merely as an external asset but as an integral part of the homestead, a sentient entity to which responsibilities are bound (Padel and Das, 2010). This scholarly work posits that the Dongria Kondh family, in their cultural ethos, functions as the fundamental and enduring ecological establishment, fostering sustainability through intergenerational cohesion and consistent ethical and legal behaviors.

In contemporary debates on sustainable futures, families are often positioned as consumers or welfare recipients rather than as governance actors. The Mainstream environmental policy in India, shaped by the Forest Conservation Act (1980) and subsequent amendments, broadly conceives conservation as a technical and bureaucratic process. Yet, parallel to this state-centerd framework, a plural legal order exists, sustained by indigenous families whose moral authority and ecological practices predate statutory law (Gadgil and Guha, 1995). This disjunction between codified governance and lived stewardship generates an epistemic gap.

The law recognizes “communities” as collective right-holders but remains silent about the familial units that practice conservation within those communities. This study seeks to bridge that gap by examining how **legal agency**, **ecological knowledge**, and **intergenerational solidarity** converge within the Dongria Kondh family to produce a resilient mode of environmental governance. First, how do family connections in indigenous communities lead to and demonstrate legal authority in protecting land and the environment? Second, how do families pass down ecological knowledge through traditions, gender-specific tasks, and spoken teachings? Third, how does intergenerational solidarity within such families translate into long-term ecological resilience and socio-legal continuity?

Addressing these questions requires an approach that combines **doctrinal legal analysis** with **interpretive sociology**, situating the Dongria Kondh's practices within India's pluralistic legal framework and the global discourse on sustainability.

The significance of these inquiries transcends the tribal boundaries. Following decades of industrial expansion in the Eastern Ghats, the Dongria Kondh have faced repeated attempts to convert their forested hills into mining zones for bauxite extraction. Their opposition to these projects culminated in the landmark Supreme Court judgment *Orissa Mining Corporation v. Ministry of Environment and Forests* (2013) 6 SCC 476 (Supreme Court of India, 2013), which recognized the primacy of the community's decision through village assemblies (gram sabhas). While the judgment is celebrated as a victory for indigenous self-determination, its more profound significance lies in how **families collectively produced legal consciousness through** elders instructing youth, mothers teaching songs that encode rights, and fathers explaining rituals of consent. These familial actions reveal that law is lived and interpreted in domestic spaces long before it appears in courts or statutes (Baxi, 2012).

The current body of literature on environmental governance seldom emphasizes the family as a focal point for analysis. Sociological studies of intergenerational solidarity often focus on care and aging (Bengtson and Roberts, 1991) but seldom extend the concept to ecological continuity. Similarly, legal anthropology has examined customary law and collective rights (Berkes, 2012; Sunder Rajan, 2013) but has not adequately explored how such norms are transmitted within kin networks. By integrating these perspectives, this research advances the argument that **families are both ecological and juridical agents**, negotiating between state law and customary moral order. Their authority stems not from formal recognition but from continuous performance, planting trees, preserving seeds, and telling stories that encode obligations to land and lineage.

Methodologically, this study adopts a doctrinal-interpretive methodology. Primary legal sources, including the Forest Rights Act (2006) and the Panchayats (Extension to Scheduled Areas) Act (1996), as well as relevant constitutional provisions, are examined alongside ethnographic texts, government reports, and NGO documentation concerning the Dongria Kondh. This combination allows an exploration of how legislation intersects with customary practices. The analysis does not treat these sources as hierarchically distinct; instead, it reads them dialogically, recognizing indigenous moral law as jurisprudence. Such an approach aligns with recent calls in socio-legal studies to decolonize the concept of law by acknowledging plural sources of authority (Merry, 2016).

This study's originality lies in the introduction of a novel triad of ideas: legal authority, ecological comprehension, and intergenerational solidarity. Legal agency denotes the capacity of family members to invoke and reinterpret norms, both statutory and customary, in pursuit of environmental justice. Ecological knowledge refers to the cumulative understanding of ecosystems sustained through everyday practice. Intergenerational solidarity captures the affective and moral ties that ensure continuity of such knowledge and its translation into collective action.

Together, these concepts form what this paper calls **familial ecological sovereignty**, a form of governance grounded in kinship ethics and spiritual reciprocity with nature.

This introductory section portrays the family as a dynamic nucleus where legal traditions, educational endeavors, and resilience converge. It sets the stage for subsequent sections that.

(a) Trace theoretical foundations of family-based environmental governance;
(b) Review the Indian legal context and its limitations;
(c) Analyze the Dongria Kondh case to illustrate how familial practices enact sustainability; and
(d) Draw policy implications for recentering families within sustainability and legal pluralism debates. The paper also critically engages with the internal tensions and risks inherent in the familial sovereignty argument—including gendered authority, dominant-lineage capture, and the household-Gram Sabha interface—in Section 5.2.

By focusing on one tribe's lived jurisprudence, the paper contributes to a broader sociological understanding of how intergenerational kinship continues to shape the moral and legal imagination of sustainable futures.

## 2 Methods: a critical interpretive review

This study adopts a critical interpretive review methodology. The approach is doctrinal and interpretive rather than empirical, owing to the absence of primary fieldwork among the Dongria Kondh. A critical interpretive review, as distinguished from a conventional systematic review, foregrounds conceptual synthesis and theory-building over exhaustive coverage, while still requiring transparent criteria for source selection (Dixon-Woods et al., 2006). Although it originated in health-sciences research, critical interpretive synthesis has since been adopted in socio-legal and wider social science scholarship as a rigorous method for theory-building from heterogeneous normative and empirical sources (Banakar and Travers, 2005).

### 2.1 Search strategy and sources

The review draws on three bodies of material. First, Indian statutory and constitutional texts: the Forest (Conservation) Act, 1980; the Panchayats (Extension to Scheduled Areas) Act, 1996; the Scheduled Tribes and Other Traditional Forest Dwellers (Recognition of Forest Rights) Act, 2006; the Biological Diversity Act, 2002; and relevant constitutional provisions. Judicial decisions were located through SCC Online, Manupatra, and Indian Kanoon. Second, peer-reviewed sociological, legal-anthropological, and political-ecology scholarship, identified through Google Scholar, JSTOR, HeinOnline, and Web of Science using search strings including “Dongria Kondh”, “Niyamgiri”, “Forest Rights Act” AND “customary law”, “legal pluralism” AND “indigenous”, “traditional ecological knowledge” AND “kinship”, and “intergenerational solidarity” AND “environment”. Third, gray literature from established sources: Economic and Political Weekly case studies, Rights and Resources Initiative (RRI) reports, Amnesty International and Survival International documentation on the Niyamgiri dispute, and Ministry of Tribal Affairs Community Forest Resource (CFR) implementation updates.

### 2.2 Inclusion and exclusion criteria

Sources were included where they (a) addressed the Dongria Kondh or comparably situated Adivasi communities in eastern India; (b) engaged substantively with legal pluralism, commons governance, kincentric ecology, or intergenerational transmission of ecological knowledge; or (c) offered doctrinal or jurisprudential analysis bearing on indigenous environmental rights in India. The temporal scope was 1980 to 2024, with earlier classics retained where doctrinally load-bearing (e.g., Ehrlich, 1936; Llewellyn and Hoebel, 1941). Sources were excluded where they were purely technical natural-science studies without a socio-legal dimension, or where scholarly provenance could not be established.

### 2.3 Rationale for case selection

The Dongria Kondh were selected because the Niyamgiri dispute has produced an unusually well-documented intersection of customary governance, statutory forest rights, and constitutional adjudication, culminating in *Orissa Mining Corporation v. Ministry of Environment and Forests* (2013) 6 SCC 476, where the Supreme Court routed the question of bauxite mining through 12 Gram Sabha consent votes under Section 6 of the Forest Rights Act. This convergence permits close examination of how familial and community institutions interact with formal legal processes. Supporting cases (Samatha; T.N. Godavarman; Subhash Kumar; M.C. Mehta v. Kamal Nath; NALSA) were selected for their doctrinal bearing on environmental rights, customary law, and collective entitlements.

### 2.4 Analytical limitations

Three limitations should be acknowledged. First, in the absence of original fieldwork, inferences about Dongria familial practice depend on an existing ethnographic record, principally Padel and Das (2010), and on broader Adivasi scholarship (Baviskar, 2003; Gadgil and Guha, 1992), which is uneven in its attention to intra-household dynamics. Claims about specific ritual, pedagogical, or deliberative practices should therefore be read as interpretively reconstructed rather than empirically verified. Second, source selection involves curatorial judgment; the author has sought to mitigate confirmation bias by engaging countervailing perspectives on the romanticisation of indigenous governance (Agrawal and Gibson, 1999) and on intra-community inequality (Sundar, 2007; Kelkar and Nathan, 1991). Third, the argument is developed through one extended case; generalization to other Adivasi groups or to non-Indian indigenous contexts is suggested but not demonstrated, and is flagged as a direction for comparative empirical work.

## 3 Theoretical framework: families as sites of law, ecology, and intergenerational solidarity

The conceptual foundation of this study rests on the proposition that families, particularly those embedded in indigenous societies, operate as micro-institutions where ecological knowledge, legal agency, and intergenerational solidarity intersect. Conventional sustainability discourse tends to locate governance within the state, market, or community but rarely attributes epistemic or juridical authority to families themselves. Yet anthropological and sociological literature reveals that kinship structures not only organize social reproduction but also mediate environmental decision-making (Ingold, 2000; Bird-David, 1999). In these frameworks, the household serves as a place for sustenance and ethical deliberation, where standards related to land, work, and mutual support are consistently discussed. In this section, the Dongria Kondh family is described as a legal and ecological institution representing legal authority, ecological understanding, and intergenerational unity.

At the outset, three terms that the literature often conflates require differentiation. *Family* in this paper refers to the co-residential household, typically a multi-generational unit of grandparents, parents, and children sharing subsistence, ritual life, and caregiving responsibilities. *Kinship* denotes the wider web of consanguineal and affinal relations (lineage, clan) that structures inheritance, marriage alliance, and ceremonial obligation but does not reside under a single roof. *Community* refers to the village or hamlet (padara) that aggregates multiple families and kin groups for collective decision-making, including the Gram Sabha when constituted under the PESA and FRA frameworks. The argument advanced here is not that the family is isomorphic with the community, but that the family is the smallest durable institution within which ecological knowledge, legal reasoning, and intergenerational obligation circulate; and that its analytic separation from the community matters because statutory law currently collapses them.

Legal agency, in the context of indigenous governance, refers to the capacity of individuals or collectives to interpret, assert, and enforce law outside formal state institutions. Legal pluralism scholarship has long established that multiple normative orders coexist within a single political system (Griffiths, 1986; Merry, 1988). For communities like the Dongria Kondh, such plurality is not a peripheral reality but the center of social life. Kin groups exercise *familial sovereignty* through ritual authority and consensus, determining access to forests, allocating cultivation plots, and conducting religious ceremonies. These decisions are legally meaningful even if they are not codified, for they establish obligations that are socially binding and morally enforceable (Benton, 2012).

The family acts as a small governing body, turning theoretical rights and responsibilities into practical actions. When elders convene to decide whether a patch of forest may be cleared, they enact law through deliberation and precedent. The collective moral reasoning guiding such acts resembles what Llewellyn and Hoebel (1941) described as the “case method” of primitive law decisions grounded in contextual fairness rather than general rules. This performative understanding of law challenges the positivist distinction between “legal” and “non-legal” domains, highlighting how kinship groups maintain normative order in ways analogous to state institutions. For the Dongria Kondh, law is inseparable from land and lineage; deciding about land is deciding about family continuity. Ecological knowledge among the Dongria Kondh is not stored in written archives but circulated through daily practice, observation, and oral instruction. The family functions as the primary educational site where environmental literacy is cultivated. Elders teach younger members the rhythms of shifting cultivation (*podu*), recognizing soil fertility through smell and texture; women identify medicinal herbs and monitor water sources; and seasonal rituals align agricultural activities with ecological cycles (Padel and Das, 2010; Gadgil et al., 1993). This pattern of knowledge transmission exemplifies what Berkes (2012) terms “traditional ecological knowledge” (TEK), a cumulative system of understanding relationships between living beings and their environment.

Within kinship pedagogy, learning is inextricably linked to emotion and memory. Songs sung during planting or harvest encode taxonomies of plants and animals; myths link species with ancestral events, creating moral obligations to protect them (Guha and Martinez-Alier, 1997). Through such practices, ecological knowledge becomes simultaneously scientific, spiritual, and social in nature (McGregor, 2014). It is also dynamic. Younger generations modify techniques in response to climatic variation, ensuring adaptability. The family thus operates as a self-renewing institution of environmental education, one that maintains the continuity of biodiversity knowledge in the absence of formal schooling systems. Recognizing this pedagogical role broadens the sociological understanding of sustainability from a policy framework to a lived epistemology sustained by affective bonds.

The third pillar of this framework, intergenerational solidarity, connects the ethical dimensions of family life with ecological resilience. Sociologists define intergenerational solidarity as the set of norms and exchanges that bind different generations within a family, encompassing emotional closeness, functional support, and value consensus (Bengtson and Roberts, 1991). In indigenous contexts, however, solidarity extends beyond human kin to include ancestors, deities, and the non-human world. The Dongria Kondh's veneration of *Niyamraja*, the mountain deity, transforms environmental protection into filial duty, an act of respect toward both ancestors and future descendants (Padel and Das, 2010). Caring for the forest equates to caring for the family, and vice versa.

This relational ontology resonates with feminist care ethics, which conceptualizes morality as emerging from networks of dependency and empathy rather than autonomous choice (Gilligan, 1982; Tronto, 1993). In the Dongria Kondh society, women's labor in seed preservation and forest gathering exemplifies this ethic of care. Their practices sustain food security and ecological balance, demonstrating that resilience is an outcome of relational rather than merely technical knowledge. Intergenerational solidarity thus constitutes a **moral economy of care**, a system through which emotional, material, and ecological responsibilities are reproduced over time. It offers a counterpoint to the atomized individualism of modern sustainability discourse by situating resilience within kinship and reciprocity.

By intertwining these three elements of legal agency, ecological understanding, and intergenerational unity, the conceptual framework of familial ecological sovereignty evolves. The term captures the overlapping spheres of authority exercised by families in governing land, transmitting knowledge, and sustaining collective identity. It diverges from conventional understandings of sovereignty as territorial control, instead emphasizing relational autonomy grounded in moral obligation and ecological intimacy. Familial ecological sovereignty is exercised not through coercion but through consent, ritual, and care. It thrives in the interstices of formal law and customary practice, offering a vernacular model of governance that aligns with the broader sociological agenda of decolonizing environmental knowledge (Escobar, 2018; Whyte, 2018).

The argument of *familial ecological sovereignty* stands at the intersection of three literatures it borrows from and one it modifies. Commons scholarship in the Ostrom tradition has long treated local resource regimes as nested institutions, with the household appearing as a largely unexamined sub-unit beneath the user group and the appropriation authority (Ostrom, 1990, pp. 88–102). The familial sovereignty argument opens that box: it asks what happens within the household that makes the community-tier commons durable across generations, and treats intergenerational knowledge transmission as a design feature rather than a background condition. Agrawal and Gibson's (1999) critique of the “mythic community” applies here with equal force to the family, and I return to their warning in Section 5.2. Salmón's (2000) notion of kincentric ecology and Kimmerer's (2013) recent extension of it describe an ethical orientation in which humans treat non-human beings as relatives. I take a narrower institutional step: the family is not only the cultural setting in which that orientation is absorbed, it is the site at which it becomes binding through ritual, inheritance, and consent to collective action. A third body of work, running through Borrows (2010) and Napoleon (2013) on indigenous legal orders, treats such orders as generated continuously through land-based practice and narrative rather than codified once and then applied. Familial ecological sovereignty, in that framing, is not an alternative to community-tier indigenous law. It is the reproductive substrate without which community-tier law has no intergenerational carrier. The household sits inside the Gram Sabha as the Gram Sabha sits inside the CFR regime, accountable upward to both, and accountable inward to its own members, including those whose voices the household does not always hear.

Conceptually, this framework extends the debate on sustainability by introducing kinship as both an analytic and normative category. It suggests that resilience arises when knowledge, authority, and care circulate within intergenerational loops rather than through impersonal institutions. For policy and law, it implies that protecting indigenous environments requires recognizing families not merely as beneficiaries but as partners in governance. In the chapters that follow, this framework will guide the analysis of the Dongria Kondh's lived jurisprudence and the ways their familial structures enact environmental ethics within India's plural legal landscape.

## 4 Legal context in India: constitutional and statutory dimensions

India's constitutional framework embodies a complex interplay between environmental protection, social justice, and cultural autonomy. Within this framework, indigenous families, particularly those of Scheduled Tribes, occupy a dual legal position: they are citizens under the modern state, yet guardians of customary norms that predate it. Understanding this legal tension is essential for situating the Dongria Kondh's ecological stewardship within the constitutional vision of sustainable development and intergenerational responsibility. The Indian legal order thus provides both the normative constraints and the emancipatory possibilities through which familial ecological sovereignty can be articulated.

The Indian Constitution, though originally silent on environmental rights, has evolved to recognize ecology as intrinsic to the right to life. The Directive Principles of State Policy (Articles 48A and 51A[g]) explicitly impose duties upon the State and citizens to protect and improve the environment and to safeguard forests and wildlife. Judicial interpretation has expanded these provisions into enforceable rights through Article 21, which guarantees the right to life and personal liberty. Landmark decisions such as Subhash Kumar v. State of Bihar (1991); Supreme Court of India (1991); M.C. Mehta v. Kamal Nath (1997) and Supreme Court of India (1997a) established that the right to a clean and healthy environment is integral to Article 21.

This constitutional jurisprudence constructs environmental protection as a public trust, positioning the state as a trustee of natural resources [see M.C. Mehta v. Kamal Nath (1997)]. However, this trustee model has often been critiqued for its centralizing tendencies. For communities like the Dongria Kondh, whose ecological relations are anchored in collective customary ownership rather than individual rights, constitutional ecology requires a plural interpretation, one that recognizes the family and community as co-trustees of nature. Such an interpretation aligns with the Supreme Court's gradual move toward recognizing indigenous environmental rights, particularly under the doctrine of sustainable development, as seen in *T.N. Godavarman Thirumulpad v. Union of India* (2002), Supreme Court of India (2002).

The most direct legislative recognition of indigenous families as ecological agents is found in the *Scheduled Tribes and Other Traditional Forest Dwellers (Recognition of Forest Rights) Act, 2006* (hereafter referred to as the Forest Rights Act or FRA). The FRA reconfigures the relationship between the state, forests, and forest-dwelling communities by acknowledging the latter's historical rights over forest land. It grants both individual and community rights, including the right to hold and live in forest land under occupation, the right to minor forest produce, and, significantly, the right to protect, regenerate, or conserve community forest resources [Section 3(1)(i)].

For the Dongria Kondh, this legislative recognition validates their long-standing practice of familial custodianship of the Niyamgiri hills. The forest is not merely an economic resource, but a sacred landscape intertwined with ancestral identity. The Supreme Court's decision in *Orissa Mining Corporation v. Ministry of Environment and Forests* (2013) (Supreme Court of India, 2013) 6 SCC 476 exemplifies this paradigm. The Court upheld the Gram Sabha's authority to determine the cultural and religious rights of the Dongria Kondh over the Niyamgiri hills, effectively transforming local familial consent into a constitutional instrument of environmental governance. The judgment's emphasis on cultural survival as a dimension of ecological protection resonates with the notion of *familial ecological sovereignty*, where kinship and spirituality form the legal grounds for environmental stewardship.

The FRA also decentralizes governance through Gram Sabhas. In many Adivasi settings, the Gram Sabha has been described as a scaled-up assembly of households bound by kinship (Padel and Das, 2010); this characterization, however, is best treated as an empirical hypothesis whose strength varies across Scheduled Areas and requires ethnographic verification. Where household-to-assembly continuity does hold, the law embeds customary ecological governance within statutory form, supporting the sociological observation that sustainability is most viable when rooted in familial and community practices (Sarin et al., 2013). Where it does not, the Gram Sabha may function as a distinct deliberative tier that aggregates plural family voices rather than reproducing any single lineage. The implementation of the FRA has in any case been uneven, revealing tensions between bureaucratic rationality and customary jurisprudence (Kumar and Kerr, 2012), and underscoring the importance of recognizing indigenous legal agency as an epistemic category rather than solely an administrative one.

The recognition of customary law in India stems from both constitutional protection and statutory accommodation. Article 13 of the Constitution excludes “customs or usages” from being voided unless they contravene fundamental rights, thereby preserving space for indigenous jurisprudence (though it must be acknowledged that courts have in some decisions construed this protection narrowly, reading Article 13(1) alongside Article 13(3)(a) in ways that limit the scope of customary immunity; the interpretive space it preserves for indigenous jurisprudence should therefore be understood as real but contested). Additionally, the Fifth Schedule provides for autonomous governance in Scheduled Areas, empowering Tribal Advisory Councils and Governors to modify laws to suit local conditions. Judicial precedent has further strengthened this autonomy. In *Samatha v. State of Andhra Pradesh* (1997b) 8 SCC 191 (Supreme Court of India, 1997b), the Supreme Court held that government land in Scheduled Areas could not be leased to non-tribals or private companies, affirming tribal ownership as a collective right essential for the cultural and economic survival of the tribes.

Customary law, however, operates not as a rigid code but as a living practice of negotiation and adaptation. Among the Dongria Kondh, disputes concerning land or forest resources are typically resolved through councils of elders, where restorative rather than punitive justice prevails. The legitimacy of such decisions stems from consensus and continuity, rather than coercion. This aligns with the concept of *living law* proposed by Ehrlich (1936), which locates the source of law in social practices rather than state enactments. In this light, the Dongria family council becomes a site of jurisprudential production, translating moral duties toward the forest into binding norms of conduct.

Indian courts have occasionally recognized such customary systems, though inconsistently. The *Limbu Customary Law* case (2010) 3 SCC 569 reiterated that customary rules may coexist with statutory law, provided they are not contrary to constitutional morality. This principle of *harmonious coexistence* underlines India's commitment to legal pluralism, a commitment that extends the sociological insight that law, like culture, is multilayered and context-specific (de Sousa Santos, 2002).

India's engagement with international norms further enriches the domestic legal context of indigenous family rights. Although India has not ratified ILO Convention No. 169, it has endorsed the United Nations Declaration on the Rights of Indigenous Peoples (United Nations, 2007), which affirms the rights of indigenous peoples to maintain and strengthen their institutions, cultures, and traditions (Article 5). UNDRIP's emphasis on collective rights over lands, territories, and resources (Article 26) aligns with the FRA's recognition of community forest rights and supports the moral legitimacy of familial custodianship of nature.

Indian jurisprudence has also internalized elements of these norms through the doctrine of constitutional compassion, as articulated in *National Legal Services Authority v. Union of India* (2014) 5 SCC 438, where the Supreme Court invoked international human rights to interpret constitutional equality expansively. Applying similar reasoning, one can argue that recognizing the Dongria Kondh family's ecological role constitutes an act of restorative justice a means of correcting historical exclusion by integrating customary environmental wisdom into constitutional morality.

The National Biodiversity Act, 2002, also operationalizes India's international obligations under the Convention on Biological Diversity. It introduces the concept of “People's Biodiversity Registers,” encouraging local communities to document and protect traditional ecological knowledge (Sections 36–41). This legislative device parallels the intergenerational transmission of environmental knowledge within Dongria families, translating familial memory into legal evidence. It symbolically bridges the domestic and international spheres by converting lived kinship practices into formal instruments of biodiversity governance.

Synthesizing these legal provisions suggests that existing doctrines and statutes can be interpreted to support the sociological notion of *familial ecological sovereignty*, even though no Indian constitutional text explicitly names the family as an ecological actor. The Constitution's environmental jurisprudence, the FRA's recognition of community rights, and the pluralist ethos of customary law can together be read to construct a normative framework in which families act as co-governors of ecological systems. This is an interpretive reading, not an established doctrine. The persistence of extractive economic policies and bureaucratic inertia continues to marginalize familial jurisdictions in practice. The Niyamgiri case illustrates both the interpretive potential and the fragility of the reading: while the judiciary affirmed the Dongria Kondh's right to religious and cultural autonomy through the Gram Sabha mechanism, subsequent administrative decisions have constrained the practical exercise of those rights.

To sustain this fragile balance, Indian environmental governance must shift from a top-down regulatory model to one of relational accountability and transparency. This approach respects the moral and ecological reasoning embedded in family practices. Embedding kinship-based governance within constitutional interpretation offers a pathway for democratizing sustainability. It situates environmental protection not as a technocratic duty, but as an ethical continuum that links generations, species, and cosmologies. This reorientation requires that policymakers and jurists view indigenous families not as subjects of conservation, but as agents of law-making, whose authority stems from their enduring connection to the land.

## 5 Discussion: intergenerational solidarity and the resilience of familial ecologies

Intergenerational solidarity serves as a vital link connecting sociology, law, and environmental ethics. It reframes sustainability not simply as the management of natural resources but as the endurance of social relationships across time. Within indigenous societies such as the Dongria Kondh, the transmission of ecological knowledge, legal norms, and spiritual obligations across generations constitutes a living model of resilience. This section discusses how familial institutions sustain environmental continuity, the challenges they face under modern developmental pressures, and the sociological implications of recognizing such practices as legitimate forms of ecological governance.

Among the Dongria Kondh, familial organization forms the architecture through which ecological memory is maintained. The family household, encompassing multiple generations, functions as a repository of experiential knowledge. Each generation contributes to and modifies this repository through learning, ritual, and adaptation. For instance, elders recount origin stories that link rivers and hills to ancestral beings, thus embedding moral obligations within geography. These narratives not only orient present conduct but also ensure that the younger generation perceives the landscape as sentient and relational (Ingold, 2000). Such mythic cartographies constitute what anthropologists describe as *cosmological mapping*, a way of encoding ecological knowledge within cultural symbolism (Descola, 2013).

This familial transmission contrasts sharply with modern educational institutions that often abstract knowledge from lived experience. In the Kondh hills, ecological knowledge is inextricably linked to subsistence practices: children learn about soil fertility, seasonal winds, and plant behavior through direct participation. This is an intergenerational pedagogy, both affective and practical, where ecological learning is embedded in familial intimacy. Studies of other tribal communities in India, such as the Baigas and the Bhils, demonstrate similar modes of transmission where land-use decisions are based on inherited patterns of observation rather than written records (Gadgil and Guha, 1992; Berkes, 2012). Such practices reveal that indigenous families operate as self-sustaining educational systems, ensuring ecological adaptability without external intervention.

The Dongria Kondh's governance of the Niyamgiri hills also illustrates how familial solidarity manifests as a legal principle. Law, in this context, is not confined to formal codification but is lived through everyday rituals and decisions. When elders resolve disputes over forest use or marriage alliances, they interpret customary law in a way that balances social harmony with ecological integrity. This process of legal reasoning mirrors the deliberative ethics of care described in feminist scholarship, where justice is achieved through relational understanding rather than abstract universality (Tronto, 1993; Gilligan, 1982).

From a sociological perspective, this conflation of kinship and law complicates conventional notions of governance. It shifts the locus of authority from distant bureaucracies to proximate moral communities. Legal anthropologists have long argued that such *vernacular law* operates through consensus rather than coercion (Merry, 1988; de Sousa Santos, 2002). Within the Dongria family council, decisions are binding not because they are enforced by external power but because they reaffirm collective identity. This form of legal agency reflects what de Sousa Santos (2002) calls *subaltern legality*, a domain where marginalized groups generate alternative legalities that coexist with and challenge state-centric law.

The recognition of such legalities within India's constitutional framework, particularly through the Forest Rights Act and the jurisprudence of *Orissa Mining Corporation v. Ministry of Environment and Forests* (2013), demonstrates a gradual shift in jurisprudence toward relational forms of justice. By treating the Gram Sabha's familial consent as a determinant of environmental decision-making, the Supreme Court effectively acknowledged that the source of legitimacy lies within the social ecology of the family. This legal pluralism offers a profound sociological insight: that families are not merely units of reproduction but institutions of governance capable of moral and ecological reasoning.

Dongria Kondh families' resilience underscores a fundamental sociological principle: resilience as a social infrastructure. Modern development policies often equate resilience with infrastructural or technological adaptation. Yet in indigenous societies, resilience arises from the continuity of social relationships that allow communities to recover from disruption without losing their moral core. The Dongria Kondh's collective resistance to bauxite mining in the Niyamgiri hills exemplifies this principle. Their mobilization drew strength not from external organizations but from familial unity, where decision-making was communal and guided by elders. Each household's commitment to protecting ancestral land was part of a larger moral economy of reciprocity and care (Padel and Das, 2010).

Interpretations of intergenerational resilience highlight the critical role of shared values over material accumulation. It is an economy of care that transcends economic rationality. Elders invest in the well-being of the young through teaching and ritual instruction, while younger members ensure the survival of elders through labor and reverence. The circulation of such obligations creates a form of social capital that underpins ecological sustainability (Putnam, 2000). When families fragment under external pressures, such as migration, displacement, or industrialization, the environmental balance they maintained also collapses. Therefore, supporting familial cohesion is not merely a cultural concern but an ecological imperative.

Despite their resilience, indigenous families face profound vulnerabilities in contemporary India. The expansion of mining, infrastructure, and industrial projects often disrupts the kinship-based ecologies that sustain environmental knowledge. Displacement fractures intergenerational ties and erases the spatial contexts in which ecological memory is reproduced. Studies of the Vedanta mining project in Odisha reveal that relocation not only leads to loss of land but also disintegration of moral economies rooted in familial relations (Padel and Das, 2010; Baviskar, 2003). The commodification of forest produce further transforms subsistence practices into market-oriented labor, undermining the ethics of reciprocity that once governed resource use.

Legally, these disruptions expose the limitations of existing frameworks. Although the Forest Rights Act and constitutional jurisprudence recognize community rights, their enforcement depends on state bureaucracy, which often fails to understand the familial basis of ecological governance. Administrative procedures, such as proof of residence or lineage documentation, are ill-suited to oral societies. Consequently, many families remain legally invisible despite their cultural and ecological contributions (Kumar and Kerr, 2012). This legal invisibility mirrors what Scott (1998) describes as the “state's legibility project,” the attempt to simplify complex local realities into bureaucratic categories. For indigenous families, such simplification translates into exclusion from both law and land. Recognizing families as agents of sustainability requires a shift from paternalistic conservation to participatory governance. Policies must move beyond treating indigenous families as beneficiaries of development schemes to acknowledging them as co-authors of ecological knowledge. This reimagining entails embedding intergenerational solidarity within environmental law and policy through three pathways.

### 5.1 Niyamgiri in longer perspective: 2003–2024

The Niyamgiri dispute pre-dates the 2013 judgment by almost a decade and continues in contested form afterwards. In 2003, Sterlite Industries (later Vedanta Resources) entered into a memorandum of understanding with the Government of Odisha to mine bauxite on the Niyamgiri hills, and the alumina refinery at Lanjigarh was approved in 2004. From 2004 onwards, Dongria Kondh families, supported by the Niyamgiri Suraksha Samiti and allied advocacy organizations, mobilized in opposition through padayatras, testimonies before statutory committees, and representations to the Ministry of Environment and Forests (Padel and Das, 2010; Amnesty International, 2010). The Saxena Committee report (Ministry of Environment and Forests, 2010) documented violations of the FRA and PESA in the clearance process, contributing to the revocation of Stage II forest clearance.

In *Orissa Mining Corporation v. Ministry of Environment and Forests* (2013) 6 SCC 476, the Supreme Court directed that the question of whether the Niyamgiri hills hosted community and religious rights be placed before the Gram Sabhas under Section 6 of the FRA. Between July and August 2013, 12 Gram Sabhas in Rayagada and Kalahandi districts unanimously rejected the mining proposal. The Ministry of Environment and Forests accordingly rejected the final clearance in January 2014. Renewed prospecting interest and state-level proposals have periodically resurfaced since 2015, and enforcement of CFR rights in the surrounding forest blocks has been uneven (Rights and Resources Initiative, 2018; Survival International, 2023). The trajectory illustrates that the judgment, while landmark, is not self-executing: its durability depends on the same intergenerational familial mobilization that produced the 2013 outcome. It should be noted that developments after 2023 fall outside the sources consulted for this review; readers seeking current information on CFR recognition and enforcement in the Niyamgiri area are directed to the Ministry of Tribal Affairs CFR portal and ongoing monitoring by the Rights and Resources Initiative.

### 5.2 Tensions, risks, and countervailing considerations

The family is not a safe moral resting place. Four difficulties with the argument I have made so far need to be stated plainly. The first is gendered authority inside the household. Adivasi families, including Dongria families, are not uniformly egalitarian; patrilineal inheritance and generational seniority shape who speaks and whose testimony counts (Kelkar and Nathan, 1991, on Jharkhandi Adivasi women and forest rights; Sundar, 2007, on Bastar). To place formal environmental authority in family councils without pausing to ask which family members are actually consulted is to risk entrenching hierarchies under the language of custom. A related difficulty is migration. Dongria and other Adivasi youth increasingly move for schooling and wage labor, and the intergenerational transmission of ecological practice, seed selection, water management, ritual timing, does not survive such absences automatically. A policy that assumes it does will fail.

The community itself is not flat. Agrawal and Gibson (1999) warned against the mythic community a quarter of a century ago, and the warning applies equally to the family: dominant lineages can convert customary legitimacy into CFR boundary claims or preferential access to minor forest produce. The literature on access and authority (Ribot and Peluso, 2003; Sikor and Lund, 2009) gives us the vocabulary for analyzing such capture; what is missing is the institutional safeguard. The fourth difficulty is specifically legal. The household and the Gram Sabha do not always speak with one voice. A claim under Section 3(1)(a) of the FRA (individual forest rights) can cut against a claim under Section 3(1)(i) (community forest resource rights), and the statute's machinery for resolving that conflict is thin. Any serious operationalization of familial ecological sovereignty has to put the Gram Sabha at the final backstop, with procedures for intra-household dissent, and with some independent check on lineage claims before they crystallize into boundaries — a function that the Forest Rights Committees constituted under Section 6(3) of the FRA are already mandated to perform and could serve as a plausible verification mechanism. Without those, the argument I have advanced is better treated as a descriptive claim about why the Dongria Kondh have held Niyamgiri for two decades than a prescriptive one about how to design the next generation of forest law.

The first move is interpretive rather than legislative. Indian environmental jurisprudence has read Article 21 expansively since Subhash Kumar v. State of Bihar (1991) and M.C. Mehta v. Kamal Nath (1997), but the familial duty of care has not been part of that expansion. There is no doctrinal bar to including it. Baxi (2010) has argued for a constitutional reading that treats human rights as emerging from concrete networks of obligation rather than abstract personhood, and indigenous kinship ethics fit that reading more comfortably than the liberal individual does. No amendment is needed; a suitably framed case before the Supreme Court would do the work.

The statutory route is more concrete. Four procedural changes, all within the existing FRA and Biological Diversity Act framework, would carry most of the work. Free, Prior and Informed Consent protocols can be embedded within the Section 6 Gram Sabha procedure, with express provision for lineage and household testimony rather than only a majoritarian show of hands. Written community protocols, of the kind now being developed under the Nagoya Protocol, can specify which household-level ecological knowledge counts for CFR decisions, reducing the state's unilateral interpretive authority. People's Biodiversity Registers, already mandated under Sections 36–41 of the Biological Diversity Act, can move from generic species lists to family-anchored micro-zoning that links specific ecological zones to named stewardship lineages. And Rule 13 of the FRA Rules, 2008, which presently privileges written documentation, can be amended to give oral history and community verification equal evidentiary weight at the claim-adjudication stage. None of these require new legislation.

The third move is the one I am least certain about. Environmental Impact Assessments (EIAs) under the 2006 EIA Notification could in principle require genuine engagement with Gram Sabhas, including rather than bypassing household and lineage testimony, but the EIA regime has too often been captured by project proponents, and procedural reform on paper has not translated into practice. A better bet may be cultural impact assessment as a distinct instrument, piloted first in Scheduled Areas and tied to specific project types such as mining and large dams, where the familial and ritual stakes are highest. Both proposals work through the existing Gram Sabha rather than around it.

Viewing the concept of familial ecological sovereignty through the lens of intergenerational solidarity enhances its empirical and normative depth. It encapsulates the idea that sovereignty is not only a political concept but also a relational one, exercised through care, memory, and moral continuity. Families, as microcosms of social order, mediate between individual and collective identities, translating ecological ethics into everyday practice. In indigenous societies, this sovereignty is exercised not through domination, but through stewardship, a recognition that human survival depends on the flourishing of non-human kin.

Sociologically, this challenges the individualistic assumptions of modern environmental governance. It aligns with theories of relational sociology, which emphasize that social reality is constituted by networks of interdependence rather than autonomous agents (Emirbayer, 1997). Within this framework, the Dongria Kondh family emerges as a *relational polity*, a collective that enacts governance through dialogue, reciprocity, and ritual. Such familial sovereignties expand the moral imagination of law and policy, urging a shift from rights-based to relationship-based sustainability. Intergenerational solidarity also offers an ethical critique of contemporary sustainability discourse. The dominant narrative of sustainable development, as codified in international instruments such as the Brundtland Report [World Commission on Environment and Development (WCED), 1987], often treats future generations as abstract entities. In contrast, indigenous families experience intergenerational responsibility as lived intimacy, the faces of children and ancestors who inhabit the same moral universe. This embodied temporality transforms sustainability from a technocratic goal into an ethical relation.

The Dongria Kondh's reverence for *Niyamraja*, the mountain deity symbolizing moral order, represents this ethic of temporal continuity. Caring for the forest is an act of reverence toward ancestors and a promise to descendants. Yet, unlike Western formulations, the Dongria practice locates these rights within family duties rather than abstract personhood. The forest is not a rights-bearing entity but a relative deserving care. This ontological shift from rights to responsibilities could enrich global environmental ethics by reintroducing the familial as a moral category.

Recognizing familial ecological sovereignty has transformative implications for sociology and law. It invites scholars to rethink the boundaries between private and public, tradition and modernity, and domestic and political spheres. Families, particularly in indigenous contexts, are not apolitical spaces but arenas of governance where ecological futures are negotiated. By foregrounding intergenerational solidarity, this research contributes to what Beck (2010) describes as “cosmopolitan sociology,” a discipline that transcends the nation-state to engage with plural ways of knowing and being.

In practical terms, strengthening familial agency can enhance resilience against climate change, resource depletion, and cultural erosion. Programmes that support community-based conservation or indigenous education could integrate family-based governance models. The sociological challenge lies in designing institutions flexible enough to accommodate such pluralities without reducing them to bureaucratic templates. Doing so requires humility and the willingness of modern law to learn from the moral intelligence of families who have sustained ecological balance for centuries.

## 6 Conclusion and policy implications: reclaiming familial ecologies for a sustainable future

The proposals that follow are normative in character: they are grounded in the descriptive and critical analysis set out in Sections 3–5, and are intended to translate that analysis into actionable directions for legal and policy reform.

Delving into the Dongria Kondh's familial ecological customs offers a profound understanding of sustainability, surpassing conventional management strategies and underscoring the family's significance in moral, legal, and environmental domains. Across the preceding sections, this study has demonstrated that families in indigenous communities are not solely responsible for biological reproduction but also uphold ecological and cultural realms through the intergenerational dissemination of knowledge, care, and authority. By placing the Dongria Kondh within the context of India's constitutional and legal frameworks, this analysis confirms that familial solidarity is not antiquated but a crucial asset in tackling the ecological challenges of the present era.

In most developmental and legal discourses, the family has been treated as a private or cultural entity, marginalized in the spheres of governance and environmental policy. However, as demonstrated by this research, families play a fundamental role in managing ecological systems. They represent a form of ecological governance based on everyday care, stewardship, and moral decision-making. The Dongria Kondh family, through its rituals of cultivation, decision-making assemblies, and reverence for Niyamraja, illustrates a distinct model of what this paper terms *familial ecological sovereignty*.

Unlike state sovereignty, which operates through control and extraction, familial sovereignty is enacted through reciprocity and restraint. It reflects a governance system grounded in kinship, memory, and moral continuity rather than bureaucracy or profit. In this way, the family transforms into an ecological establishment where coexistence ethics are not just discussed but also implemented. This shift encourages sociologists, policymakers, and jurists to move past the distinction between “modern” and “traditional,” acknowledging that sustainability may lie in the enduring nature of inherited relationships.

In India, the prevailing environmental governance model continues to be characterized by extraction-oriented practices and a high degree of centralization. Policies often focus on resource productivity or carbon offsets as measures of success, neglecting the important ethical relationships necessary for maintaining ecological equilibrium. This disconnect has marginalized indigenous families whose ecological reasoning does not conform to bureaucratic logics. Reintegrating family-based governance into environmental policy, therefore, requires a paradigm shift from *resource management* to *relational governance*.

Relational governance prioritizes the quality of social and ecological relationships over quantitative outputs. It demands that legal and administrative systems recognize kinship-based custodianship as a legitimate form of authority. For instance, Environmental Impact Assessments (EIAs) could be redesigned to include structured consultations with family councils or lineage groups, rather than token community hearings. Likewise, the *Forest Rights Act, 2006*, could institutionalize family-based monitoring mechanisms for community forest resources, providing statutory protection to those who have sustained ecological continuity for generations (Sarin et al., 2013; Kumar and Kerr, 2012).

This realignment is in harmony with the foundational principles outlined in India's constitution. The Directive Principles and the expanding interpretation of Article 21 offer ample scope for integrating intergenerational obligations into environmental policy. Judicial innovation could further interpret the right to life as encompassing familial duties toward ecological preservation, thereby humanizing constitutional environmentalism (Baxi, 2010). Such an approach would shift environmental governance from technocratic paternalism to a moral partnership between the state and the family.

Another crucial policy implication concerns the integration of familial ecological knowledge into formal education and legal literacy initiatives. Indigenous environmental knowledge systems are often dismissed as anecdotal or unscientific; yet they embody centuries of empirical observation, adapted to local ecologies (Gadgil et al., 1993). Formal curricula could incorporate modules on indigenous environmental ethics and traditional ecological knowledge (TEK), not as folklore but as legitimate epistemologies.

At the same time, legal education and environmental law training should engage with customary jurisprudence. Future policymakers and judges must understand that indigenous families are not passive recipients of law but active interpreters of justice, shaping legal outcomes. Legal and governance courses could incorporate case studies of the Dongria Kondh and similar communities to demonstrate how family-based decision-making generates sustainable results in real-life situations. Such interdisciplinary education could nurture a generation of professionals capable of bridging the epistemic gap between statutory law and lived law.

Furthermore, community-based schools could be supported to function as intergenerational learning centers, where elders transmit ecological knowledge to youth while also engaging with modern environmental science. This convergence between familial and formal education would institutionalize intergenerational solidarity within public policy, ensuring that sustainability is embedded in both pedagogy and practice (Berkes, 2012).

Recognition, if it comes, has to be paired with protection. Indigenous families in Adivasi India are collective and oral; the administrative machinery built to recognize them is individualist and written. That gap is where rights evaporate. Reform of Rule 13 of the FRA Rules, 2008 is overdue: oral testimony and community verification should count as primary, not supplementary, evidence at the claim stage. Cultural impact assessment in Scheduled Areas would close a parallel gap at the project-approval stage, where familial cohesion and ritual practice are not currently part of what an Environmental Impact Assessment considers. But the safeguards set out in Section 5.2 apply with full force here. Without gender and youth representation, without grievance redress for household dissent, and without some independent check on lineage claims, recognition becomes a route for the strongest lineage in the village to absorb authority the state used to wield. That is the risk this paper takes most seriously.

Additionally, constitutional jurisprudence could embrace a *relational reading of rights*, interpreting Article 21 in conjunction with Article 51A(g) to acknowledge the mutual obligations between human and ecological communities. The Supreme Court's evolving environmental jurisprudence already hints at such an approach, as seen in *Orissa Mining Corporation v. Ministry of Environment and Forests* (2013) and *T.N. Godavarman Thirumulpad v. Union of India* (2002). Enhancing this relational understanding could lead to the institutionalization of the concept that environmental rights are not solely individual entitlements but also familial responsibilities.

While the Dongria Kondh context is uniquely rooted in the Niyamgiri hills, the principles emerging from their practices carry global significance. Across the world, indigenous and local families maintain ecological continuity through kinship-based systems of governance, whether among the Maori of Aotearoa, the Sami of Scandinavia, or the Quechua of the Andes. These systems articulate what Escobar (2018) describes as “pluriversal design,” the coexistence of multiple ontologies of sustainability. Recognizing familial ecological sovereignty thus contributes to a global sociology of environmental pluralism, challenging the homogenizing tendencies of global governance models.

At the international level, India could draw upon the *United Nations Declaration on the Rights of Indigenous Peoples* (United Nations, 2007) to advocate for policies that integrate family-based ecological governance into sustainable development frameworks. The idea of intergenerational solidarity, emphasized in this paper, resonates with global discussions on the rights of future generations and the importance of ethical caregiving. By supporting indigenous family models, India could establish itself as a pioneer in decolonizing sustainability conversations, showing that resilience is rooted in relationships rather than strict regulations.

The sociological implications of acknowledging families as pivotal agents of sustainability hold profound significance. It challenges the division between public and private spheres that underpins modern sociology and law. The Dongria Kondh family exemplifies the *relational turn* in social theory, an orientation that views society as an evolving web of interdependence rather than a collection of autonomous actors (Emirbayer, 1997).

From an ethical standpoint, this recognition prompts a transition from anthropocentric to kincentric ethics, expanding the moral community to encompass not only human generations but also the natural world (Cullinan, 2011). The forest, in this worldview, is not a resource to be managed but a relative to be nurtured and cared for. Such ethics can re-inspire contemporary sustainability debates by restoring emotional and moral dimensions often excluded from technocratic policymaking. This reaffirms the family's essential role as a guiding principle for ethical environmental practices.

In conclusion, this paper advances a sociological and legal argument for repositioning families, particularly indigenous ones, as foundational actors in the pursuit of sustainability. Through the lens of the Dongria Kondh, it demonstrates that familial solidarity is a source of both ecological resilience and legal agency. The intergenerational transmission of knowledge, moral values, and customary law forms the invisible infrastructure that sustains biodiversity and cultural continuity.

Reimagining law and policy through the prism of *intergenerational jurisprudence of care* can transform sustainability from a developmental slogan into a lived ethic. It compels institutions to respect the slow, relational, and deeply human processes through which families cultivate ecological harmony.
